# Complete genome sequence of *Pediococcus acidilactici* A40, a bacterium with biocontrol and plant growth-promoting properties

**DOI:** 10.1128/MRA.00530-23

**Published:** 2023-08-14

**Authors:** Christian Vargas, Daniel Bautista, Hugo Jimenez, Mauricio Soto-Suarez, Silvia Restrepo, Carolina Gonzalez, Paola Zuluaga

**Affiliations:** 1 Corporación Colombiana de Investigación Agropecuaria, AGROSAVIA, Centro de Investigación Tibaitatá, Bogotá, Colombia; 2 Universidad de Los Andes, Bogotá, Colombia; University of Rochester School of Medicine and Dentistry, Rochester, New York, USA

**Keywords:** *Pediococcus acidilactici*, biocontrol, plant growth promoting, hybrid sequence assembly, genome mining

## Abstract

We report the complete genome assembly of *Pediococcus acidilactici* A40, a bacterium with biocontrol and plant growth-promoting properties, obtained from Colombia.

## ANNOUNCEMENT

*Pediococcus acidilactici* is a Gram-positive bacterium with beneficial use in a wide range of fields (1 - 3); in the case of *P. acidilactici* A40, reports presented by several works (4 - 7) demonstrated the potential of *Pediococcus* sp. A40 strain as a biocontrol agent by inhibiting pathogen growth, inducing plant disease-response marker genes, and promoting plant growth in cape gooseberry and tomato when the pathogens *Fusarium oxysporum* and *Ralstonia solanacearum* were present either individually or together in a co-inoculation.

A40 strain was isolated from oat silage from a Colombian Andean Highland geographical region. The strain was inoculated in De Man-Rogosa-Sharpe broth and incubated at 37°C for 24 h. Two-milliliter aliquots of log-phase active cultures from this broth were used to isolate genomic DNA.

DNA was extracted using DNeasy UltraClean Microbial Kit (MoBio, Carlsbad, CA). The bacterial DNA was sequenced using second- and third-generation technologies, MiSeq (Illumina) and MinION (Oxford Nanopore), respectively. For MiSeq sequencing, genomic libraries were initially prepared with the Nextera XT (Illumina) with 250-bp paired-end reads. For MinION sequencing, ONT libraries were generated using a Ligation-Sequencing kit (SQK-LSK109), according to the manufacturer’s instructions (Oxford Nanopore Technologies). The long-fragment buffer was used to enrich long DNA fragments of >3 kb and then was loaded to a MinION-R9.4.1 flow cell (FlO-MIN106) for 24 h using MinKNOW-v2.0 with a min_qscore-7-filter. FAST5 files containing the raw Nanopore signal data were converted to FASTQ format in real time using Guppy-v3.3.0. Porechop-v0.2.4 was used to trim barcode and adapter sequences (using default parameters).

The genome was assembled *de novo*. Illumina reads were quality assessed using FastQC-v11.7 (8), and low-quality reads were removed using Trimmomatic-v0.32 (9). For Nanopore reads, default parameters were used for all of the following software, low-quality reads and reads smaller than 1000 bp were removed using Filtlong-v0.2.0 (10). Then, via Trycycler-v0.5.0 (11), genome consensus was automated using Flye-v2.9.2 (12), Canu-v2.3 (13) Miniasm + Minipolish-v0.3/v0.1.3 (14), and Raven-v1.8 (15) assemblies. Pairwise circularization of the assemblies was also performed using Trycycler-v0.5.0. The consensus assembly was corrected using Nanopore reads by Medaka-v1.8 (16). Finally, the assembly was polished with the short reads using Pilon-v1.24 (17), and the quality was evaluated by BUSCO-v4.0.6 (18). The complete genome sequence was deposited in GenBank under the accession number SUB13514853.

**Fig 1:**
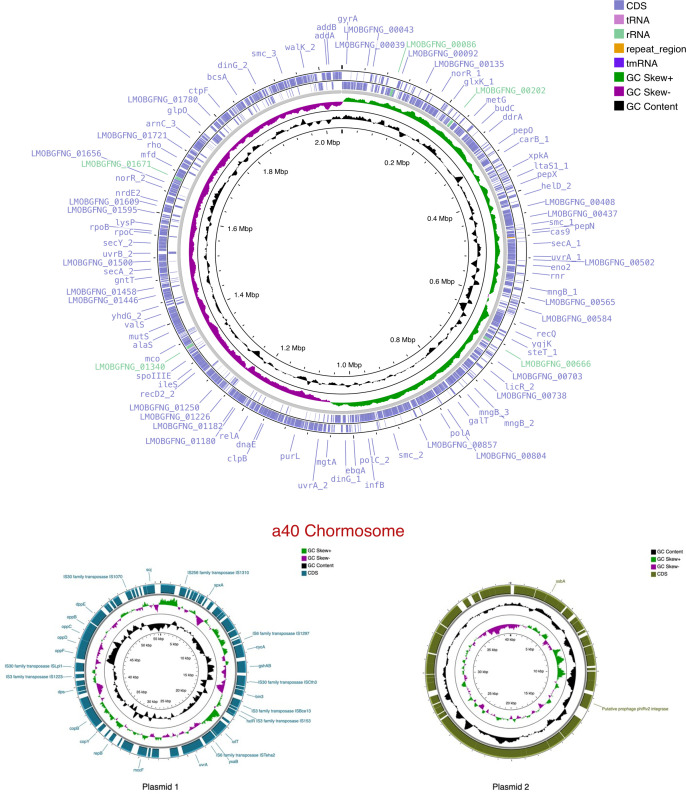
The consensus genome of lactic acid bacterium A40 and its two predicted plasmids. The consensus chromosome is circular, with 2,135,637 bp and two plasmids, one of 55,557 bp and the other one of 40,123 bp. Sequences were annotated with Prokka and show the GC percentage and bias. Hypothetical proteins are not labeled. The chromosome shows a comparison with *Pediococcus acidilactici* sequences. A total of 2,096 coding sequences (CDs) were predicted with different databases: RASTtk, Prokka, and InterProScan. All annotations gave similar CDs, ribosomal, and transfer RNA counts. Phylogenetic analysis of the 100 core genes by BcgTree indicated that the A40 strain is close to *P. acidilactici* (19). Additionally, this information was corroborated using MyTaxa and average nucleotide identity (ANI) analysis by FastANI confirming its location in *P. acidilactici* species (<0.05) (20, 21). This study provides the complete assembly of a *P. acidilactici* strain from the American continent, which may be an input for future comparative genomic studies to understand the high adaptive and functional diversity of individuals of this species.

The N50 length of the Nanopore reads was 2,040,036  bp after polishing, and the consensus assembly allowed us to obtain a genome with a 2% higher fine consistency, as well as to reduce contamination from 5.8% to 0.2% (99.0). The final genome of A40 consisted of a circular genome of 2,135,637 bp, one main chromosome, and two plasmids, one of 55,557 bp and the other one of 40,123 bp with a coverage of approximately 20× and an average GC content of 42% (Fig. 1).

## Data Availability

The described genome assembly is available in GenBank under BioSample accession number SAMN29548482. Strain A40 strain used in this work was collected under the framework collection permit no. 1466 from 2014 of AGROSAVIA and registered in the National Collections Registry (RNC129) of Colombia.
